# Linkage to and retention in chronic care among patients diagnosed with hypertension, diabetes, or HIV in DIMAMO PHRC clinics, South Africa

**DOI:** 10.1371/journal.pgph.0005362

**Published:** 2026-02-05

**Authors:** Motlatso Elias Letshokgohla, Cairo Bruce Ntimana, Eric Maimela

**Affiliations:** 1 Department of Public Health, University of Limpopo, Polokwane, South Africa,; 2 DIMAMO Population Health Research Centre, University of Limpopo, Polokwane, South Africa; 3 Department of Public Health, Walter Sisulu University, Eastern Cape, South Africa; Tribhuvan University Institute of Medicine, NEPAL

## Abstract

Chronic conditions such as hypertension, diabetes mellitus, and HIV continue to be significant contributors to morbidity and mortality in sub-Saharan Africa, including South Africa. This study aims to quantify the proportion of patients diagnosed with hypertension, diabetes, and/or HIV who are successfully linked to and enrolled in chronic care services at DIMAMO PHRC clinics. The study employed a quantitative, cross-sectional analytical design using routinely collected clinic data covering a six-month period. The research was conducted at the DIMAMO PHRC, which serves as a Health and Demographic Surveillance System. The study population was composed of patients diagnosed with hypertension, diabetes, and HIV who were aged 18 years and above. Simple random sampling was used to select the study participants. Data was analyzed using SPSS. Chi-square tests were used to compare proportions among groups. Logistic regression was used to determine the factors associated with retention to care. The proportion of individuals diagnosed with hypertension was 28.9% in both sexes, with significantly more females being hypertensive compared to males (30.9% vs 22.8%, p = 0.001). The proportion of patients retained in the linkage-to-care intervention without interruptions for six months was highest among those diagnosed with diabetes at 34.8%, followed by those diagnosed with hypertension, HIV, and both hypertension and diabetes at 29.5%, 24.8%, and 10.9%, respectively. Regression analysis showed that retention in care without gaps longer than six months was significantly associated with age, gender, and diagnosis (single chronic conditions or combined). This study identified patterns of enrolment and retention in care among patients diagnosed with chronic conditions at DIMAMO PHRC. Findings reveal that retention was highest among patients with single conditions and lowest in those with comorbid hypertension and diabetes. These trends suggest potential areas for targeted interventions to improve linkage and continuity of care, particularly among older adults and those with multiple conditions.

## Introduction

Chronic conditions such as hypertension, diabetes mellitus, and HIV remain significant contributors to morbidity and mortality in sub-Saharan Africa, including South Africa [1–3]. The prevalence of chronic illnesses is still rising, especially in marginalized and rural areas, despite improvements in diagnostic tools and the availability of efficient therapies [4]. The integration of chronic disease care, especially for non-communicable diseases (NCDs) and HIV, has become an essential strategy in strengthening primary health care and improving health outcomes [1,5]. Integrating care for chronic illnesses, particularly HIV and non-communicable diseases (NCDs), has emerged as a crucial tactic for enhancing primary healthcare and improving patient outcomes [1,6]. In this context, knowing how people are linked to and remain in care after receiving a diagnosis is essential for guiding initiatives within the health system [6].

Linkage to care, defined as the timely engagement with the health system following a confirmed diagnosis, is a crucial step in the cascade of care [7]. However, a large number of individuals with diabetes or hypertension do not regularly participate in long-term care programs, which increases the possibility of problems and hospitalizations [8,9]. Comparably, even though the Universal Test and Treat (UTT) policy has significantly improved the HIV treatment continuum, there are still gaps in ensuring that patients start and continue antiretroviral therapy (ART) [10,11]. These disparities could be caused by factors such as healthcare access, stigma, service delivery challenges, and socioeconomic barriers.

The DIMAMO Population Health Research Centre (PHRC), located in the Capricorn District of Limpopo Province, offers a unique platform for studying the dynamics of chronic disease management in a rural South African context. Through its network of primary healthcare clinics, DIMAMO PHRC provides longitudinal data on patient health, treatment adherence, and service utilization. This presents an opportunity to assess the proportion of patients who, upon diagnosis with hypertension, diabetes, or HIV, are effectively linked to and enrolled in ongoing care within the local health system. The aim of this study was to assess the proportion of patients diagnosed with hypertension, diabetes, or HIV who were linked to and retained in chronic care, and to identify factors associated with retention in care in the DIMAMO PHRC clinics. By identifying trends and gaps in care enrolment, the findings will provide evidence to guide improvements in service delivery and support the integration of care for chronic diseases. Ultimately, this work will contribute to strengthening primary healthcare systems and enhancing patient outcomes in rural South African communities.

## Methods

### Study design

The study focused on hypertension, diabetes, and HIV due to their high burden and integration within existing care programs at DIMAMO PHRC. Other chronic conditions were excluded due to their lower prevalence and incomplete data in the study setting. The study used a quantitative research approach with a cross-sectional analytical design. Although patient visit records covered a six-month period, the available dataset did not include repeated measures suitable for longitudinal modelling; therefore, the analysis is presented cross-sectionally. This design allowed comparison of retention patterns across diagnosis groups based on recorded engagement during the six-month follow-up window, providing early insights that will guide future extended follow-up analyses.

### Study setting

The study was conducted within the DIMAMO Population Health Research Centre (PHRC), located in the Capricorn District of Limpopo Province, South Africa. DIMAMO PHRC operates as a Health and Demographic Surveillance System (HDSS) covering a population of ~100,000 people across several rural villages. In addition to community-based demographic surveillance, DIMAMO PHRC supports a network of eight primary health care clinics that provide routine chronic care services for hypertension, diabetes, and HIV. These clinics differ from standard government facilities in that they are embedded within the HDSS platform, enabling more systematic data collection, longitudinal patient tracking, and integration with demographic information.

The linkage-to-care programme refers to the structured process through which patients diagnosed with chronic conditions are connected to continuous treatment and monitoring at PHRC-affiliated clinics. Enrolment occurs once a patient receives a confirmed diagnosis, either at the clinic or via referral from mobile outreach services and community-based screening campaigns. At enrolment, patients are registered in the clinic’s chronic disease register, issued a patient-held health record, and scheduled for follow-up visits. While outreach and health promotion activities raise community awareness of the programme, the majority of participants self-present to clinics after diagnosis rather than being actively traced from the community.

All services provided through this programme are free of charge under South Africa’s public health system. Patients do not pay consultation fees, and essential medications for hypertension, diabetes, and HIV are provided at no cost. This reduces direct financial barriers to accessing care and facilitates participation once patients are linked to the health system

### Selection criteria

All patients diagnosed with either hypertension, diabetes, or HIV on ART attending clinics within the DIMAMO PHRC study area were included in the current study. Although HIV–NCD multimorbidity is conceptually central to understanding integrated chronic care, these cases were excluded from the present analysis due to substantial and systematic gaps in their documentation across clinics. HIV status was routinely captured in HIV registers, while hypertension and diabetes were recorded separately in NCD registers, and the two datasets were not consistently cross-referenced. Consequently, more than one-third of patients with confirmed HIV had missing or unverified NCD status, and approximately 40% of individuals with hypertension or diabetes lacked corresponding HIV information. For patients with potential multimorbidity, diagnosis fields were frequently incomplete or contradictory across registers. Including this group would therefore introduce major non-differential misclassification bias, resulting in distorted prevalence estimates and unreliable regression outputs. Their exclusion reflects a data integrity constraint rather than a conceptual choice, and the limitation is explicitly acknowledged. Although excluded from regression analyses, clinic records suggested that a substantial proportion of patients attending DIMAMO PHRC clinics likely experienced HIV–NCD multimorbidity. However, a reliable descriptive or comparative analysis of this group was not feasible due to inconsistent cross-recording across HIV and NCD registers. Consequently, the present analysis focuses on patients with clearly documented single conditions or hypertension–diabetes comorbidity, and findings should be interpreted as reflecting early linkage and retention patterns within these analytically robust groups rather than the full spectrum of chronic multimorbidity

### Participants and sampling

Our study included patients with hypertension, diabetes, and HIV who were 18 years or older. We used simple random sampling to select participants, following the “Taro Yamane Formula” to determine the minimum sample size per health facility with a 5% sampling error and a 10% non-response rate. Out of 8 clinics in the DIMAMO HDSS, we enrolled around 3916 individuals in linkage to care. However, about 185 individuals were excluded because they were under 18 years old. Of the remaining 3731 individuals, we randomly selected 1696 participants across the eight clinics (S1 Fig) (S1 Table and S2 Table).

### Data collection

The data used to assess acceptability focused on objective measures of behavior as indicators of acceptability, specifically patients’ dropout rates, all-cause discontinuation, and withdrawal rates. Retention outcomes were derived from clinic visit records recorded over a six-month period. For those who dropped out of the linkage to care intervention, efforts were made to ascertain the reasons for their discontinuation through telephone calls, as all participants’ contact numbers were available at the clinic and in the database for linkage to care. The participants were asked for their consent to answer questions about their participation in the linkage to care program. The acceptability survey was then administered by a trained research assistant who had knowledge of the project. The survey took approximately ten to twenty minutes to complete using the data collection tool adopted from Sekhon et al. [12], which was translated into the local language, Sepedi. The survey was conducted only in Sepedi, the primary local language, as it is widely spoken and understood in the study area.

The data for this study were collected between 20 January and 30 June 2024. Patient information was extracted from routine clinic registers and electronic databases within the DIMAMO PHRC Health and Demographic Surveillance System (HDSS). The database is continuously updated by trained clinic data clerks as part of routine service delivery monitoring, with basic consistency checks performed during entry.

To ensure data quality, information extracted from clinic registers was cross-checked against corresponding electronic HDSS records to verify patient identifiers, diagnosis categories, and visit dates. Where discrepancies were detected, verification was conducted with the clinic data clerk, and the paper-based register was used as the reference source. Missing or ambiguous entries (e.g., incomplete dates or unclear diagnosis coding) were flagged and excluded from analysis. Routine internal data validation procedures at DIMAMO PHRC include monthly reconciliation between clinic and HDSS datasets to identify duplicates or gaps in reporting. Internal validation procedures were conducted monthly, involving reconciliation of clinic paper registers with HDSS electronic datasets to identify discrepancies, duplicates, and missing entries. These checks were performed by two independent data clerks and verified by the HDSS data manager. Although no external audit was conducted due to resource constraints, this internal two-tier validation approach enhances the reliability of the data used for analysis. The full set of survey questions is provided in the Supplementary Material (S1 Questionnaire).

Data completeness was assessed before analysis. Records with missing or inconsistent key identifiers (age, sex, diagnosis, or clinic visit dates) were first flagged and cross-checked against HDSS electronic records. When discrepancies could not be resolved, the affected records were excluded. Across the eight clinics, 9.4% (n ≈ 157) of extracted records were excluded due to missing visit dates or incomplete diagnosis classification, and an additional 4.8% (n ≈ 80) were excluded due to mismatched identifiers or duplicated entries. Missing data on secondary variables (e.g., marital status) were handled using listwise deletion for regression models, as imputation was not feasible given the categorical nature of the dataset and the lack of auxiliary variables. This approach ensured internal consistency and prevented misclassification bias.

### Outcome definitions

**Linkage to care**: documented enrolment of a patient in chronic care services at a PHRC clinic following a confirmed diagnosis of hypertension, diabetes, or HIV. This included registration in the clinic’s chronic care register and at least one clinical visit for the relevant condition after diagnosis

**Retention in care**: continued engagement in chronic care following linkage, assessed as the absence of any gaps longer than six months between recorded clinic visits. Patients with gaps >6 months were classified as “not retained.

Retention was operationalized as a binary indicator because the available clinic records contained only the dates of the first and most recent visits within the six-month follow-up window. Intermediate visit timestamps, missed appointment logs, and re-engagement records were not consistently documented across facilities. As such, it was not possible to distinguish temporary interruptions, delayed medication pickups, or re-engagement episodes from permanent disengagement. Multi-state modelling or survival/time-to-event approaches require chronological visit-level data, which were not available in the current dataset. The binary outcome, therefore, reflects the most reliable and consistently recorded retention measure across all eight clinics.

### Statistical analysis

Data was analyzed using SPSS version 28. Categorical variables were presented in percentages and frequencies. Chi-square tests were used to compare proportions among groups, including comparisons of retention rates across different diagnoses. Logistic regression was used to determine the factors associated with retention in care with and without Gaps >6 Months. The dependent variables were retention to care with and without Gaps >6 Months, while the independent variables were age groups (Categorized as, 18–34 years, ≥ 35 years, 35–44 years, 45–54 years,55–64 years, and ≥ 65 years), marital status (categorized as, married, single, divorced, and widowed), gender (Males and females), and diagnosis (Hypertension, diabetes, HIV, and hypertension plus/ and diabetes). These variables were chosen because they were consistently recorded across all clinic registers. Other potentially important covariates, such as socioeconomic status, education level, employment status, distance to clinic, and disease severity, were not available in the current dataset and could not be included in the regression models. As such, the associations reported should be interpreted cautiously, as they may partially reflect unmeasured contextual or structural factors rather than direct causal relationships. Future analyses using the expanding DIMAMO PHRC data will incorporate these variables to improve model adjustment and explanatory power. Logistic regression was selected due to the binary nature of the outcome variable (retention with vs without gaps >6 months). Independent variables were selected based on prior evidence and theoretical relevance. No data transformation was needed beyond categorical coding of variables such as age and diagnosis. A *p*-value of less than 0.05 was considered statistically significant.

Missing data were handled using complete case analysis. No statistical imputation was applied, as the available variables did not support robust estimation of missing covariate values. The proportion of missingness per variable was generally low (<5%), except for diagnosis classification within multimorbidity cases, which exceeded 35% and was therefore excluded from modelling. Sensitivity checks were performed to ensure that excluded records did not differ systematically from included records in terms of age or sex distributions, and no significant differences were observed. This reduces the likelihood of selection bias stemming from missing data.

More granular retention outcomes, such as multi-state transitions (e.g., engaged → interrupted → re-engaged) or duration-to-disengagement models, were considered but not feasible given the structure of the data. Clinic registers did not capture the sequence or timing of all patient visits, preventing estimation of time-dependent hazards or trajectories. For this reason, the binary classification (gap ≤ 6 months vs. > 6 months) was selected as the only outcome that could be reliably derived from available records.

The statistical analysis was cross-sectional, using retention status determined from clinic visit records over a six-month period. The dataset did not contain time-varying covariates or repeated measures required for longitudinal modelling. However, due to the short follow-up period and the lack of time-varying covariates, full longitudinal modeling (e.g., mixed-effects or time-to-event analyses) was not feasible. Future analyses will incorporate extended follow-up (12–24 months) and include time-dependent covariates to better capture dynamic changes in care engagement over time

### Ethical statement

The study was conducted in accordance with the Declaration of Helsinki. Ethical approval was obtained from the Turfloop Research and Ethics Committee (TREC), with reference number TREC/1763/2023: PG. Permission to conduct the study was also granted by the Limpopo Department of Health at both provincial and district levels. The privacy of research participants was protected by using pseudonyms or letters of the alphabet to ensure anonymity. The participants were informed that participation is voluntary and they can also withdraw at any point, and those who wanted to take part in this study gave consent by signing the consent form.

## Results

Table 1 shows the proportion of patients enrolled in linkage to care intervention; the proportion of those diagnosed with hypertension was 28.9% in both sexes, with significantly females being hypertensive as compared to males (30.9% vs 22.8%, p = 0.001). The proportion of males with diabetes (35.8%) was higher than the proportion of females with diabetes (30.5%). The proportion of those diagnosed with both hypertension and diabetes was significantly higher in males than in females.

**Table 1:** The proportion of patients enrolled in the linkage to care intervention.

Diagnosis	Both sexes	Males (n = 413)	Females (n = 1260)	P-value
	% (95% CI)	% (95% CI)	% (95% CI)	
Hypertension	28.9 (26.8 – 31.2)	22.8 (18.9 – 27.1)	30.9 (28.5 – 33.6)	0.001
Diabetes	31.8 (29.6 – 34.1)	35.8 (31.3 – 40.6)	30.5 (27.9 – 33.1)	0.042
HIV	23.3 (21.3 – 25.3)	21.5 (17.8 – 25.8)	23.8 (21.5 – 26.2)	0.345
Hypertension and Diabetes	16.0 (14.3 – 17.9)	19.9 (16.3 – 23.9)	14.8 (12.9 – 16.8)	0.014

The enrollment of patients into linkage-to-care interventions varied notably by sex, age group, and diagnosis. In females, hypertension was most common in the 35–44 age group (29.5%), while in males the highest proportion was seen in the ≥ 65 group (44.7%) (p = 0.006). For diabetes, prevalence increased with age in both sexes, reaching 33.6% in older females (≥65) and 39.9% in older males. However, this was statistically significant only among females (p < 0.001). In terms of HIV, females’ enrollment decreased steeply with age, from 44.0% in the 18–34 age group to just 1.7% in those aged 65 and above. Conversely, male enrollment increased with age, peaking at 38.2% in the 45–54 age group, before decreasing in older age (p < 0.001). For patients with both hypertension and diabetes, both sexes showed an age-associated increase in linkage, with males showing a slightly higher proportion in the 65 + age group compared to females (53.7% vs 44.6%, p < 0.001). Notably, in both sexes, no patients under 45 years were enrolled for comorbid hypertension and diabetes (Table 2).

**Table 2:** The proportion of patients enrolled in the linkage to care intervention stratified by age groups and gender.

Females (n = 1260)	Age groups					
Diagnosis	18 - 34 years n (%)	35 - 44 years n (%)	45 - 54 years n (%)	55 - 64 years n (%)	≥65 years n (%)	P-value
Hypertension	77 (19.7)	115 (29.5)	101 (25.9)	26 (6.7)	71 (18.2)	<0.001
Diabetes	53 (13.8)	103 (26.8)	55 (14.3)	44 (11.5)	129 (33.6)	<0.001
HIV	132 (44.0)	103 (34.3)	49 (16.3)	11 (3.7)	5 (1.7)	<0.001
Hypertension and Diabetes	0 (0.0)	0 (0.0)	29 (15.6)	74 (39.8)	83 (44.6)	<0.001
Males (n = 413)	Age groups					
Diagnosis	18 - 34 years n (%)	35 - 44 years n (%)	45 - 54 years n (%)	55 - 64 years n (%)	≥65 years n (%)	P-value
Hypertension	16 (17.0)	7 (7.5)	18 (19.2)	11 (11.7)	42 (44.7)	0.006
Diabetes	12 (8.1)	34 (22.9)	27 (18.2)	16 (10.8)	59 (39.9)	0.069
HIV	18 (20.2)	33 (37.1)	34 (38.2)	1 (1.1)	3 (3.4)	<0.001
Hypertension and Diabetes	0 (0.0)	0 (0.0)	10 (12.2)	28 (34.2)	44 (53.7)	<0.001

Retention without interruptions was highest among patients with diabetes (34.8%), followed by those with hypertension (29.5%), HIV (24.8%), and hypertension plus diabetes (10.9%), at p-value 0.011. A similar trend has been noted in patients retained in linkage-to-care having interruptions within six months, as the majority were in those diagnosed with diabetes at 30.8% followed by those diagnosed with hypertension, HIV, and both with hypertension and diabetes at 28.7%, 22.7% and 17.7% respectively, at p-value 0.011 (Table 3).

**Table 3:** The proportion of patients enrolled and retained in the linkage to care intervention stratified by diagnosis.

Diagnosis	No gaps >6 months 420 (25.1%)	P-value for trend (diagnosis)	Not consistent >6 months 1253 (74.9%)	P-value for trend (diagnosis)
	% (95% CI)		% (95% CI)	
Hypertension	29.5 (25.3 – 34.1)	0.011	28.7 (26.3 – 31.3)	0.011
Diabetes	34.8 (30.3 – 39.5)		30.8 (28.3 – 33.4)	
HIV	24.8 (20.9 – 29.1)		22.7 (20.5 – 25.2)	
Hypertension and Diabetes	10.9 (8.3 – 14.3)		17.7 (15.7 – 19.9)	

The proportion of patients enrolled and retained in linkage to care intervention stratified by age groups and gender. In females, those with no gaps >6 months were most represented in the 35–44 age group (33.1%), followed by the 18–34 group (24.9%) and 65+ (18.3%). However, those with inconsistent care were many in the 65 + age group (24.5%) and 35–44 years (22.7%), indicating that older females were more likely to have gaps in care, while younger and middle-aged females were more consistently engaged (p < 0.001). Among males, the highest proportion retained without gaps was in the ≥ 65 group (25.6%), but this same age group also had the largest proportion with inconsistent care (38.4%) (p < 0.001) (Table 4).

**Table 4:** The proportion of patients enrolled and retained in the linkage to care intervention stratified by age groups and gender.

	Females (n = 1260)		Age groups			
18 - 34 years n (%)	35 - 44 years n (%)	45 - 54 years n (%)	55 - 64 years n (%)	≥65 years n (%)	P-value
No gaps >6 months	84 (24.9)	112 (33.1)	52 (15.4)	28 (8.3)	62 (18.3)	<0.001
Not consistent >6 months	178 (19.3)	209 (22.7)	182 (19.7)	127 (13.8)	226 (24.5)	<0.001
	Males (n = 413)		Age groups			
	18 - 34 years n (%)	35 - 44 years n (%)	45 - 54 years n (%)	55 - 64 years n (%)	≥65 years n (%)	P-value
No gaps >6 months	9 (10.9)	28 (13.9)	18 (21.9)	6 (7.3)	21 (25.6)	<0.001
Not consistent >6 months	37 (11.2)	46 (13.9)	71 (21.5)	50 (15.1)	127 (38.4)	<0.001

Retention in care without gaps longer than six months was significantly associated with age, gender, and diagnosis. Compared to the 18–34 age group, older patients had lower odds of consistent care, with the lowest retention observed among those aged 55–64 (OR=0.4; 95% CI: 0.3–0.7) and ≥65 years (OR=0.5; 95% CI: 0.4–0.8), both statistically significant at p < 0.001. Males were also significantly less likely to maintain consistent care than females (OR=0.7; 95% CI: 0.5–0.9; p < 0.05). In terms of diagnosis, individuals with hypertension (OR=1.7), diabetes (OR=1.8), or HIV (OR=1.8) had higher odds of consistent retention compared to those with both hypertension and diabetes. In contrast, for patients with inconsistent care (gaps >6 months), the odds increased significantly with age. Individuals aged 55–64 were 2.3 times more likely (OR=2.3; 95%CI:1.4–3.5) and those ≥65 years had OR=1.8 (95%CI:1.3–2.6) to experience inconsistent care, compared to the 18–34 reference group. Males were also more likely than females to have gaps (OR=1.5; 95% CI: 1.1 – 1.9). Marital status did not show statistically significant associations with retention patterns (Table 5).

**Table 5:** Logistic Regression of Factors Associated with Retention to Care with and Without Gaps >6 Months.

Variables	No gaps >6 months OR,95%CI	Not consistent >6 months OR,95%CI
Age groups		
18–34 years	Ref	Ref
≥ 35 years	0.80 (0.74 – 0.87)***	1.24 (1.15 – 1.35)***
18 – 34 years	Reference (1)	Reference (1)
35 – 44 years	1.26 (0.92 – 1.75)^a^	0.78 (0.57 – 1.08)^a^
45–54 years	0.64 (0.44 – 0.92)*	1.56 (1.09 – 2.24)*
55 – 64 years	0.44 (0.28 – 0.68)***	2.25 (1.44 – 3.49)***
≥ 65 years	0.54 (0.39 – 0.76)***	1.84 (1.31 – 2.59)***
Gender		
Female	Reference (1)	Reference (1)
Male	0.68 (0.51 – 0.89)*	1.48 (1.13 – 1.94)*
Marital status		
Married	Reference (1)	Reference (1)
Single	1.44 (0.72 – 2.84)^a^	0.69 (0.35 – 1.38)^a^
Divorced	1.63 (0.31 – 8.54)^a^	0.62 (0.12 – 3.23)^a^
Widowed	0.54 (0.12 – 2.54)^a^	1.85 (0.39 – 8.65)^a^
Diagnosis		
Hypertension plus diabetes	Reference (1)	Reference (1)
Hypertension	1.66 (1.14 – 2.42)*	0.60 (0.41 – 0.88)*
Diabetes	1.83 (1.26 – 2.64)**	0.55 (0.39 – 0.79)**
HIV	1.76 (1.219 – 2.59)**	0.57 (0.438 – 0.84)**

Values are reported as odds ratios (95%CI); * significant at p < 0.05; ** significant at p < 0.005, *** Significant at p < 0.001; ^a^ not significant.

## Discussion

The study analysed 1673 participants who were enrolled in the linkage to care intervention, with the majority of the participants being females as compared to males. The most prevalent condition among participants was diabetes (31.8%), followed by hypertension (28.9%), HIV (23.3%), and comorbid hypertension and diabetes (16.0%). These findings represent observed distributions rather than causal patterns and suggest that diabetes and hypertension were the most frequently documented conditions in the DIMAMO HDSS. The proportion of people living with HIV (23.3%) reflects the ongoing combined burden of infectious and non-communicable diseases in many low- and middle-income countries [13,14]. In addition, the presence of comorbid hypertension and diabetes at 16.0% highlights a need for integrated care models that can effectively address multimorbidity [15].

This analysis extends previous HDSS-based studies by simultaneously examining linkage and retention across HIV, hypertension, and diabetes within the same rural health system. Whereas most prior HDSS analyses have focused on single conditions or specific age groups, this study used a cross-sectional analysis based on follow-up records covering six months [16,17]. This methodological approach enables comparison across conditions within a unified health system framework, providing early insights into how integration challenges differ between infectious and non-communicable disease programs. In doing so, the study provides comparative insights into early linkage and retention across major chronic disease programmes within a rural South African HDSS platform. The findings should therefore be interpreted as early signals of potential patterns, not as evidence of causal relationships.

The proportion of hypertension was highest among females aged 35–44 years (29.5%) and among males aged 65 years and older (44.7%). These findings represent age- and sex-related differences in reported hypertension, not differences attributable to biological or behavioural causes. The prevalence of diabetes rose progressively with age, peaking in the 65 + age group, where it was 33.6% among females and 39.9% among males, indicating a higher burden among older men compared to women. HIV decreased with age among females but increased with age among males, peaking at 45–54 years before declining in older men. For patients with both hypertension and diabetes, both sexes showed an age-associated increase in linkage, with males showing a slightly higher proportion in the 65 + age group compared to females. These trends are consistent with patterns reported in other studies and likely reflect differences in health-seeking behaviour and service utilization rather than causal relationships. These findings are in alignment with the existing literature, highlighting the importance of age and sex in the prevalence of hypertension, diabetes, and HIV [18–21].

Our findings also align with a growing body of evidence on integrated chronic care in sub-Saharan Africa, where linkage and retention patterns vary across conditions and demographic groups [18,21,22]. Similar to reports from South Africa and Uganda, we observed higher retention among patients with single conditions compared to those with multimorbidity, underscoring the challenges of delivering integrated care in resource-limited settings [22,23]. These results contribute to ongoing debates about the feasibility of integrated HIV–NCD care models, highlighting the need for tailored approaches that address multimorbidity, particularly in older adults. The exclusion of HIV–NCD multimorbidity in this analysis further underscores how fragmentation of routine clinic registers constrains both service integration and the evaluation of multimorbid care in rural low- and middle-income country health systems. By situating our study within this broader literature, we demonstrate both the promise and the complexity of integrated chronic care in rural African contexts. Importantly, these patterns should not be interpreted as causal but as associations that may reflect underlying differences in service accessibility, disease awareness, and health-seeking behaviours across population groups. Because key contextual factors such as socioeconomic status, transport availability, and disease severity were not captured in the dataset, some of the observed patterns may reflect unmeasured confounding rather than true differences in behaviour or clinical engagement.

Furthermore, this study’s comparative design across multiple chronic conditions offers a distinct contribution to the growing HDSS literature. While prior studies have reported on disease-specific linkage or retention, few have simultaneously explored multimorbidity within the same analytic framework [24,25]. The present findings, therefore, offer both contextual and methodological novelty by revealing how disease type, gender, and comorbidity status interact to shape retention trajectories in a real-world, integrated primary health care setting.

The proportion of patients retained in linkage-to-care intervention without interruptions for six months was higher among those diagnosed with diabetes at 34.8%, followed by patients diagnosed with hypertension, HIV, and those with both hypertension and diabetes at 29.5%, 24.8%, and 10.9%, respectively. Regression results showed that single chronic conditions (hypertension, diabetes, or HIV) were associated with higher odds of consistent retention in care compared to those with comorbid hypertension and diabetes. A similar pattern was observed in patients retained in linkage-to-care with interruptions within six months, with those diagnosed with diabetes at 30.8%, followed by patients diagnosed with hypertension, HIV, and those with both hypertension and diabetes at 28.7%, 22.7%, and 17.7%, respectively. The lower retention rates observed among patients with both hypertension and diabetes may reflect the challenges of managing multiple chronic conditions in settings where integrated care is limited [26,27]. In many rural areas, patients are required to attend separate clinics or appointments for different conditions, increasing both the financial and logistical burden of care [28]. This fragmented approach may contribute to disengagement, particularly for patients facing transport costs, long waiting times, or competing responsibilities [28]. As this group represents one of the most clinically vulnerable populations, strengthening integrated models of chronic care delivery is critical to reduce attrition and improve outcomes [27]. In contrast with the findings of the present study, previous studies reported that HIV, both combined with comorbid hypertension and diabetes, had higher retention in care [22,23]. The inconsistencies between the findings of the present study and the existing literature may be due to the duration of the linkage to care interventions; the present study used six months, while other studies used 12 months. Another reason may be due to the different sociodemographic factors of the participants.

The findings of the present study reported that older females were more prone to experiencing gaps in care, whereas younger and middle-aged females showed more consistent engagement. In agreement with the findings of the present study, Gumede and colleagues [29] reported that older women often face challenges that can lead to interruptions in care. The majority of older women look after their grandchildren, which can reduce their ability to prioritize their health-seeking behavior [29]. These findings underscore the importance of designing tailored interventions that support older women with caregiving responsibilities and make healthcare services responsive to their needs. Although comparable trends have been documented elsewhere [30,31], the study adds value by focusing on a rural Limpopo population with high multimorbidity, where evidence has been scarce. By identifying that retention is lowest among patients with comorbid hypertension and diabetes, and that older women face unique barriers linked to caregiving roles, we provide context-specific insights that can inform targeted interventions [32,33]. This evidence is directly relevant for strengthening integrated NCD-HIV programs in primary health care, particularly in resource-constrained rural districts [34]. These associations likely reflect underlying social and structural realities, including gendered caregiving roles, economic dependency, and limited-service flexibility, that shape retention behaviours beyond individual-level determinants.

Moreover, older males demonstrated higher engagement levels but also a higher likelihood of inconsistent care, suggesting a decline in consistency in the oldest age group compared to females. This indicates that while many older men remained engaged, they were also the group most vulnerable to interruption. Indicating that while older males were more likely to be in care, they were disproportionately affected by inconsistent engagement, showing a more pronounced decline in care consistency in the oldest age group compared to females. These findings corroborate the study by Tshuma et al. [35], who reported that while older men may initially engage with healthcare services, sustaining consistent care remains a challenge, highlighting the issues of societal norms and delayed health-seeking behaviour. The findings of the present study, therefore, suggest that while older adults, particularly men, are likely to enter care, they face greater challenges in maintaining long-term continuity, leading to higher odds of inconsistent retention.

Although age and sex were significantly associated with retention outcomes, these relationships should be interpreted considering potential residual confounding. Key contextual determinants of chronic care engagement in rural South Africa, such as socioeconomic status, household income, employment, distance to clinic, transport availability, and disease severity, were not available in the routine clinic dataset and could not be adjusted for in the models. These factors are unevenly distributed across age and sex groups and may partially explain the observed associations. For example, older adults and women may face greater transport constraints, caregiving responsibilities, or financial dependency, which could contribute to higher rates of care interruption independent of age or biological sex. Similarly, men who remain in care may represent a more health-motivated subgroup, potentially inflating apparent sex differences in retention. As a result, the age- and sex-stratified findings reported here should be understood as reflecting both individual characteristics and unmeasured structural and socioeconomic conditions rather than direct causal effect.

In rural South Africa, practical barriers such as transport difficulties, long clinic wait times, and competing livelihood responsibilities also disproportionately affect older adults who depend on family support [36,37]. These contextual and health system constraints, such as human resource shortages, poor service integration, and inadequate patient follow-up mechanisms, further shape retention outcomes. These patterns highlight that retention challenges are not only demographic but embedded within broader structural and cultural realities. Addressing societal norms around masculinity and promoting health-seeking behavior through targeted campaigns can improve consistency in care [38].

Beyond individual demographic and psychosocial factors, system-level barriers within rural primary health care settings are likely to play a major role in shaping retention outcomes**.** Health service fragmentation, where HIV, hypertension, and diabetes care operate in parallel rather than as fully integrated services, has been widely documented to increase patient burden, reduce care efficiency, and weaken continuity in sub-Saharan Africa [25,27,34]. Chronic care platforms in rural South Africa also face structural constraints such as staff shortages, long waiting times, limited clinic space, and inconsistent follow-up systems, which hinder regular clinic attendance [28,37]. Weak referral and patient-tracking mechanisms further impede timely re-engagement of patients who miss appointments, disproportionately affecting multimorbid patients who require more complex coordination across services [22,25]. In addition, transportation barriers and long distances to clinics, common in rural districts, reduce accessibility for older adults and low-income households [36]. These health-system limitations likely contribute substantially to the retention patterns observed in this study, underscoring that differences across age, gender, and diagnosis groups may reflect broader structural inequities rather than individual behaviours alone

For patients with inconsistent care (gaps >6 months), the odds increased significantly with age. Individuals aged 55–64 were 2.3 times more likely (OR=2.3; 95%CI:1.4–3.5) and those ≥65 years had OR=1.8 (95%CI:1.3–2.6) to experience inconsistent care, compared to those aged 18–34. These findings are in agreement with previous studies that indicate that an increase in age is significantly associated with inconsistent engagement in care, particularly among older adults [39,40]. Taken together, these results suggest associations between demographic factors and retention that are mediated by the structure of rural health systems, accessibility challenges, and social responsibilities. Targeted interventions such as caregiver support for older women and male-focused health-seeking campaigns may help mitigate these risks [41].

It is also important to interpret these findings within the context of missing socioeconomic and access-related information, which may influence retention outcomes in ways not accounted for in the current models. The findings have several implications for policy and practice. For older adults, targeted support could include transport subsidies or community-based medicine delivery to reduce access barriers, as well as caregiver support services to ease competing household responsibilities. Flexible clinic hours may also benefit older women and working-age adults [42]. For patients with multiple chronic conditions, integrated service models that synchronize appointments and treatment refills across conditions would reduce the burden of multiple clinic visits [43]. Task-shifting to community health workers for follow-up and adherence support could further strengthen continuity of care [44]. At a broader system level, these interventions must be embedded within structural reforms that address the persistent fragmentation of chronic care services, workforce shortages, and underinvestment in primary healthcare infrastructure [45]. Strengthening health information systems, ensuring regular medication supply chains, and enhancing coordination between HIV and NCD programs are essential to improve service integration and continuity. At the system level, strengthening electronic patient tracking and patient reminder systems may help identify and re-engage those at risk of disengagement earlier. By linking these recommendations to systemic constraints, the findings highlight that improving retention requires both patient-level interventions and structural reforms that build a more responsive, equitable, and integrated health system in rural South Africa [46,47].

Residual confounding remains an important consideration when interpreting these findings**.** Although age, gender, and diagnosis were included in the models, several important contextual determinants, such as socioeconomic status, education level, employment status, household income, clinic distance, transport access, and disease severity, were not available in the routine dataset. These variables are well-established predictors of chronic-care engagement in rural South Africa, and their absence may partly explain some of the observed differences across age and gender groups. For example, individuals living further from clinics or with lower socioeconomic status may have been disproportionately represented among those with gaps in care. Similarly, disease severity may influence retention differently across conditions, but this could not be assessed. The associations reported here should therefore be interpreted cautiously, as they may reflect underlying structural and social barriers rather than direct causal effects. Incorporating these variables in future analyses will allow more comprehensive adjustment and strengthen interpretability

While prior HDSS-based analyses have typically examined single diseases or used disease-specific cohorts, this study makes three distinct contributions. First, it compares linkage and early retention (six-month follow-up) across *three* major chronic conditions (hypertension, diabetes and HIV) within the *same* rural HDSS-supported network of eight primary-care clinics, allowing direct, within-system contrasts that reduce inter-study heterogeneity. Second, the study applies a transparent, reproducible operational definition of retention (absence of gaps >6 months) and documents the data-cleaning, cross-checking and exclusion criteria used where register completeness was limited, an explicitly pragmatic approach that reflects real-world routine-data constraints and that we fully describe to help others replicate or extend our methods. Third, by using random sampling across all clinics and reporting clinic-level data quality procedures, the analysis provides unusually detailed information about routine HDSS clinic data integrity and early retention dynamics in a rural South African setting. Together, these features provide methodological and programmatic novelty: they move beyond single-disease descriptions to generate comparative, system-level evidence about where and for whom early disengagement is most likely, and they make explicit how routine HDSS clinic data can (and cannot) be used for early retention surveillance and targeted service improvement.

### Study limitations and strengths of the study

The current study focused on patients diagnosed with certain conditions who were receiving services at primary health care clinics. Consequently, it was challenging to obtain complete data about all newly diagnosed individuals, especially those not actively seeking care or unreachable by typical outreach methods. This limitation may introduce selection bias, as the participants in the study might not fully represent the wider population affected by the relevant diseases. The six-month follow-up window may not fully capture long-term retention patterns for lifelong conditions such as HIV and diabetes. Although the programme follow-up period spanned six months, the available dataset did not include repeated measurements of covariates or visit-level clinical information. As a result, the analysis is cross-sectional, and the study does not model temporal changes or time-varying predictors. The binary measure used in this analysis does not capture important distinctions such as temporary versus permanent disengagement, late medication pickups, or patterns of re-engagement. These care trajectories are clinically and programmatically meaningful, but their identification requires complete visit histories, which were not available in routine clinic registers during this study period. Consequently, our findings reflect a conservative summary of retention status at six months rather than true longitudinal care patterns. Future work at DIMAMO PHRC will incorporate a fully timestamped visit history, enabling multi-state modelling and survival analysis to characterize patient trajectories better. Although many studies report retention at 12 months, this study assessed retention over six months [48,49]. This shorter interval was selected because it aligned with the available follow-up data during the study window and allowed for early assessment of retention dynamics in a rural South African context where such evidence is limited [50,51]. While this choice limits direct comparability with longer-term studies, it provides important baseline insights into early patterns of engagement and identifies groups most vulnerable to early disengagement. Although the study adopted a cross-design, the analysis was based on follow-up records covering a six-month period but constrained by the limited time horizon and lack of time-varying covariates. As data matures, more robust longitudinal modeling will be undertaken. The exclusion of patients with HIV–NCD multimorbidity due to incomplete and inconsistent documentation across HIV and NCD clinic registers. Multimorbidity is central to chronic care delivery in low- and middle-income countries, and excluding this group limits the generalizability and conceptual completeness of the findings. Patients with coexisting HIV and NCDs often experience more complex care pathways, higher treatment burden, and greater vulnerability to disengagement than those with single conditions. Consequently, the retention patterns reported here may represent conservative estimates of the challenges faced within fully integrated chronic care settings.

However, inclusion of this group in the present analysis would have introduced substantial misclassification bias, given the high proportion of missing and contradictory diagnosis information across registers. This exclusion, therefore, reflects a data integrity constraint rather than a conceptual decision. Ongoing data harmonisation efforts at DIMAMO PHRC will enable future analyses to explicitly incorporate HIV–NCD multimorbidity and provide a more comprehensive assessment of integrated chronic care outcomes.

This population is central to integrated chronic care in LMICs, and their exclusion reduces generalizability. However, including them with the current level of missingness and conflicting information would have introduced substantial bias. In addition, key contextual variables such as socioeconomic status, education level, employment, distance to clinic, and disease severity were not captured in the present dataset. The absence of these variables likely resulted in residual confounding, meaning that some of the observed associations between age, sex, and retention may reflect unmeasured socioeconomic, access-related, and disease-severity factors rather than true differences in care engagement behaviour. Future work at DIMAMO PHRC includes this group as data completeness improves, to better reflect the realities of multimorbidity in primary healthcare settings, and will also integrate these variables to allow more comprehensive and adjusted modeling of retention determinants. Although comprehensive internal validation procedures were undertaken, the absence of an independent external audit remains a limitation. Furthermore, the use of complete case analysis may lead to underestimation of uncertainty, although missingness was largely random with respect to age and sex. Future data harmonization efforts at DIMAMO PHRC, including unified clinic registers and automated cross-checking systems, will reduce missingness and allow for more sophisticated handling of incomplete data.

The study focused exclusively on quantitative measures, without qualitative insights into structural barriers, patient experiences, or provider perspectives that could contextualize retention patterns. Ongoing work at DIMAMO PHRC aims to address this gap through qualitative investigations that complement the present findings. Nevertheless, the study offers valuable insights into patient retention in care, with a significant sample size of participants diagnosed with diabetes, hypertension, or HIV from eight different clinics, followed for six months after enrolment in the linkage to care intervention. This prospective cohort study was adequately powered and demonstrated good participant retention.

## Conclusion

This study identified patterns of enrolment and retention in care among patients diagnosed with chronic conditions at DIMAMO PHRC. Findings reveal that retention was highest among patients with single conditions and lowest in those with comorbid hypertension and diabetes. Given their particularly low retention, patients with both hypertension and diabetes should be considered a critical priority group for interventions. Improving integrated models of care that address multiple chronic conditions simultaneously will be essential to enhance linkage and continuity of care, particularly among older adults and those with multimorbidity. In addition to reaffirming global patterns, our work highlights context-specific challenges and opportunities in rural South Africa, such as the need for differentiated models for comorbid patients and gender-sensitive interventions for older women. However, as these findings are derived from observational data, they should be interpreted as associations rather than causal relationships. These findings point to the need for integrated, patient-centred service delivery models that address the unique barriers faced by older adults and multimorbid patients. Importantly, improving retention requires system-wide reforms, such as strengthening health workforce capacity, integrating chronic care delivery, and addressing transport and socioeconomic barriers to create an enabling environment for sustained patient engagement. Policy actions such as expanding caregiver support, providing transport assistance, and embedding multimorbidity care into routine PHC services could play a vital role in improving retention. The findings extend beyond descriptive reporting and offer practical entry points for health system improvement.

The study contributes novel evidence on the early dynamics of linkage and retention within an HDSS-supported rural health system, emphasizing differences across disease categories and patient profiles. By integrating multiple chronic conditions within a single analytic framework, it provides a more comprehensive understanding of how multimorbidity and health system organization intersect to influence continuity of care. This approach represents a methodological and interpretive advancement in the study of chronic disease management within rural South African HDSS settings, offering new empirical insights to guide integrated service delivery and health system reform.

Given the cross-sectional nature of the analysis, these findings reflect associations with retention status at six months rather than longitudinal patterns of engagement. Future work at DIMAMO PHRC will extend follow-up to 12–24 months and incorporate qualitative research to identify context-specific solutions. These will support the development of practical, evidence-based interventions to strengthen chronic care retention in rural South African settings.

## Supporting information

S1 Questinaire Questinaire.
(DOCX)

S1 Table Number of participants per clinic linked to care. (DOCX)

S2 Table Sample Size per clinic. (DOCX)

S1 Fig Participants selection. (DOCX)
