# Supplementary material for: Linkage to and retention in chronic care among patients diagnosed with hypertension, diabetes, or HIV in DIMAMO PHRC clinics, South Africa
Source: PLOS Glob Public Health. 2026 Feb 5;6(2):e0005362. doi: 10.1371/journal.pgph.0005362 (PMC12875484; doi:10.1371/journal.pgph.0005362)
Supplement: S1 Questinaire — Questinaire. (DOCX) [file pgph.0005362.s001.docx]

## **DATA COLLECTION TOOL FOR PATIENTS LINKAGE TO CARE INTERVENTION**

## **ACCEPTABILITY FOR THE INITIAL ROUND OF COHORT**

Please answer these questions that deal with your reactions to the proposed Linkage to Care program. Circle the number that best describes your reactions.

| Patient Unique Identifier | |  | | Educational level | No schooling | |  | Marital status | Single | |  |
| --- | --- | --- | --- | --- | --- | --- | --- | --- | --- | --- | --- |
| Age in years | |  | |  | Primary | |  |  | Married | |  |
| Gender | | **Male** | **Female** |  | Secondary | |  |  | Divorced | |  |
| Medical condition | |  | |  | Tertiary | |  |  | Widowed | |  |
| Are you on treatment of this condition | | **Yes** | **No** | For how long have you been on treatment (Duration in months or years) | |  | | | | | |
|  |  |  |  | How often do you collect you medication | | **Monthly** | | **Quarterly** | **Six-monthly** | | |
|  | Focus Area | | | Set questions | | | | | **Response** | | |
|  | Affective attitude | | | - 1. I am glad that I was asked to participate in the linkage to care intervention | | | | | **Yes** | **No** | |
|  |  |  |  | - 1. I have enjoyed the discussions with the linkage to care facilitator | | | | | **Yes** | **No** | |
|  |  |  |  | - 1. I have appreciated the activities suggested in the program for linkage to care intervention | | | | | **Yes** | **No** | |
|  |  |  |  | - 1. I have enjoyed working with care companions in the linkage to care intervention | | | | | **Yes** | **No** | |
|  |  | | |  | | | | | **Yes** | **No** | |
|  | Burden | | | - 1. It has been easy and effortless to have the session with the linkage to care intervention facilitator | | | | | **Yes** | **No** | |
|  |  |  |  | - 1. It has been easy to find time to participate in the linkage to care intervention | | | | | **Yes** | **No** | |
|  |  |  |  | - 1. The length of the linkage to care intervention sessions was not too long | | | | | **Yes** | **No** | |
|  |  | | |  | | | | | **Yes** | **No** | |
|  | Perceived effectiveness | | | - 1. The linkage to care intervention has helped me to comply to my treatment | | | | | **Yes** | **No** | |
|  |  |  |  | - 1. The linkage to care intervention has helped me to focus much on my health outcomes | | | | | **Yes** | **No** | |
|  |  |  |  | - 1. I feel that I have achieved the goals set together with the linkage to care intervention facilitator to adhere to treatment | | | | | **Yes** | **No** | |
|  |  |  |  | - 1. I feel my health is better now compared to when I started the linkage to care intervention | | | | | **Yes** | **No** | |
|  |  | | |  | | | | | **Yes** | **No** | |
|  | Intervention coherence | | | - 1. It has been easy to understand how this linkage to care intervention can help me | | | | | **Yes** | **No** | |
|  |  |  |  | - 1. I feel that I have received enough information about the linkage to care intervention | | | | | **Yes** | **No** | |
|  |  | | |  | | | | | **Yes** | **No** | |
|  | Opportunity costs | | | - 1. I have changed my schedule to be able to participate in the intervention sessions | | | | | **Yes** | **No** | |
|  |  |  |  | - 1. I have spent much time with my health care workers due to the participation in the linkage to care intervention | | | | | **Yes** | **No** | |
|  |  | | |  | | | | | **Yes** | **No** | |
|  | Self-efficacy | | | - 1. I am confident I can continue with the new habits discussed with the linkage to care intervention facilitator in my daily life to comply to treatment | | | | | **Yes** | **No** | |
|  |  | | |  | | | | | **Yes** | **No** | |
|  | Ethicality | | | - 1. The activities in this linkage to care intervention have fitted well with how I want to live my life | | | | | **Yes** | **No** | |
|  |  |  |  | - 1. The possibility for support from others besides healthcare providers is important for me | | | | | **Yes** | **No** | |

## **SEDIRIŠWA SA KGOBOKETŠO YA DATHA SA KGOKAGANO YA BALWETŠI GO KAMOGELO YA TSENOGARE YA TLHOKOMELO BAKENG SA TIKOLOGO YA MATHOMO YA SEHLOPHA**

Hle araba dipotšišo tše tšeo di lebanego le dikarabelo tša gago lenaneong leo le šišintšwego la Kgokaganyo le Tlhokomelo. Dira sediko palo yeo e hlalosago dikarabelo tša gago gabotse.

| Sešupo sa Moswananoši sa Molwetši | |  | Boemo bja thuto | | **Ga se ka tsena sekolo** | | |  | | Boemo bja lenyalo | | **Single** | | |  | |  |
| --- | --- | --- | --- | --- | --- | --- | --- | --- | --- | --- | --- | --- | --- | --- | --- | --- | --- |
| Mengwaga | |  |  |  | **Sekolo sa tlasana** | | |  | |  |  | **Nyetšwe** | | |  | |  |
| Bong | | **Monna** | **Mosadi** |  | | **Sekolo sa bobedi** | | |  | |  | | **Ba hlalane** | | |  | |
| mohuta wa bolwetši | |  |  | | **Sekolo sa boraro** | | |  | |  | | **Mohlologadi** | | |  | |  |
| Na o kalafong ya boemo bjo | | **Ee** | **Aowa** | O na le nako ye kaakang o le kalafong (Duration in months or years) | | |  | | | | | | | | | | |
|  |  |  |  | Ke ga kae o go kgoboketša dihlare | | | **Kgwedi le kgwedi** | | | | **Kotara le kotara** | | **Kgwedi ye tshela ka kgwedi** | | | | |
|  | Focus Area | | Set questions | | | | | | | | | **Ee** | | | | |  |
|  | Boemo bja kgopolo bjo bo nago le maikwelo | | - 1. Ke thabela gore ke kgopetšwe go tšea karolo kgokaganong ya tsenogare ya tlhokomelo | | | | | | | | | **Ee** | | **Aowa** | | |  |
|  |  |  | - 1. Ke thabetše dipoledišano le kgokagano le mofahloši wa tlhokomelo | | | | | | | | | **Ee** | | **Aowa** | | |  |
|  |  |  | - 1. Ke lebogile mediro yeo e šišintšwego lenaneong la kgokagano le tsenogare ya tlhokomelo | | | | | | | | | **Ee** | | **Aowa** | | |  |
|  |  |  | - 1. Ke thabetše go šoma le bagwera ba tlhokomelo kgokaganong ya tsenogare ya tlhokomelo | | | | | | | | | **Ee** | | **Aowa** | | |  |
|  |  | |  | | | | | | | | | **Ee** | | **Aowa** | | |  |
|  | Morwalo | | - 1. E bile bonolo ebile go se na maitapišo go ba le sešene yeo e nago le kgokagano le mofahloši wa tsenogare ya tlhokomelo | | | | | | | | | **Ee** | | **Aowa** | | |  |
|  |  |  | - 1. Go bile bonolo go hwetša nako ya go kgatha tema kgokaganong le tsenogare ya tlhokomelo | | | | | | | | | **Ee** | | **Aowa** | | |  |
|  |  |  | - 1. Bolelele bja kgokagano le dithulaganyo tša tsenogare ya tlhokomelo e be e se botelele kudu | | | | | | | | | **Ee** | | **Aowa** | | |  |
|  |  | |  | | | | | | | | | **Ee** | | **Aowa** | | |  |
|  | Boitshwaro bja boitshwaro | | - 1. Kgokagano le tsenogare ya tlhokomelo e nthušitše go obamela kalafo ya ka | | | | | | | | | **Ee** | | **Aowa** | | |  |
|  |  |  | - 1. Kgokaganyo le tsenogare ya tlhokomelo e nthušitše go tsepelela kudu dipoelong tša ka tša maphelo | | | | | | | | | **Ee** | | **Aowa** | | |  |
|  |  |  | - 1. Ke ikwa gore ke fihleletše dinepo tšeo di beilwego mmogo le kgokagano le mofahloši wa tsenogare ya tlhokomelo go kgomarela kalafo | | | | | | | | | **Ee** | | **Aowa** | | |  |
|  |  |  | - 1. Ke kwa maphelo a ka a le kaone bjale ge ke bapetšwa le ge ke thoma kgokagano le tsenogare ya tlhokomelo | | | | | | | | | **Ee** | | **Aowa** | | |  |
|  |  | |  | | | | | | | | | **Ee** | | **Aowa** | | |  |
|  | Go šoma gabotse mo go lemogwago | | - 1. Go bile bonolo go kwešiša ka fao kgokagano ye le tsenogare ya tlhokomelo e ka nthušago ka gona | | | | | | | | | **Ee** | | **Aowa** | | |  |
|  |  |  | - 1. Ke ikwa gore ke amogetše tshedimošo ye e lekanego mabapi le kgokagano le tsenogare ya tlhokomelo | | | | | | | | | **Ee** | | **Aowa** | | |  |
|  |  | |  | | | | | | | | | **Ee** | | **Aowa** | | |  |
|  | Kgokagano ya tsenogare | | - 1. Ke fetotše lenaneo la ka gore ke kgone go tšea karolo dithutong tša tsenogare | | | | | | | | | **Ee** | | **Aowa** | | |  |
|  |  |  | - 1. Ke feditše nako ye ntši le bašomi ba ka ba tlhokomelo ya maphelo ka lebaka la go kgatha tema kgokaganong le tsenogare ya tlhokomelo | | | | | | | | | **Ee** | | **Aowa** | | |  |
|  |  | |  | | | | | | | | | **Ee** | | **Aowa** | | |  |
|  | Go ikgona | | - 1. Ke na le tshepo ya gore nka tšwela pele ka mekgwa ye mefsa yeo e ahlaahlilwego le kgokagano le mofahloši wa tsenogare ya tlhokomelo bophelong bja ka bja letšatši le letšatši go obamela kalafo | | | | | | | | | **Ee** | | **Aowa** | | |  |
|  |  | |  | | | | | | | | | **Ee** | | **Aowa** | | |  |
|  | Ditshenyagalelo tša sebaka | | - 1. Mešomo yeo e lego kgokaganong ye le tsenogare ya tlhokomelo e dumelelane gabotse le ka fao ke nyakago go phela bophelo bja ka ka gona | | | | | | | | | **Ee** | | **Aowa** | | |  |
|  |  |  | - 1. Kgonagalo ya thekgo go tšwa go ba bangwe ntle le baabi ba tlhokomelo ya maphelo e bohlokwa go nna | | | | | | | | | **Ee** | | **Aowa** | | |  |

## **DATA COLLECTION TOOL FOR PATIENTS LINKAGE TO CARE INTERVENTION ACCEPTABILITY FOR FOLLOW UP LONGITUDINAL COHORTS**

Please answer these questions that deal with your reactions to the proposed Linkage to Care program. Circle the number that best describes your reactions.

| Patient Unique Identifier |  | | Educational level | No schooling | |  | Marital status | Single |  |
| --- | --- | --- | --- | --- | --- | --- | --- | --- | --- |
| Age in years |  | |  | Primary | |  |  | Married |  |
| Gender | **Male** | **Female** |  | Secondary | |  |  | Divorced |  |
| Medical condition |  | |  | Tertiary | |  |  | Widowed |  |
| Are you on treatment of this condition | **Yes** | **No** | For how long have you been on treatment (Duration in months or years) | |  | | | | |
|  |  |  | How often do you collect you medication | | **Monthly** | | **Quarterly** | **Six-monthly** | |
| **Affective attitude**  *How an individual feels about the linkage to care intervention* | | | Did you like the linkage to care intervention   \| Strongly dislike \| Dislike \| No opinion \| Like \| Strongly like \| \| --- \| --- \| --- \| --- \| --- \| \| **1** \| **2** \| **3** \| **4** \| **5** \|   Did you like the linkage to care intervention   \| Strongly dislike \| Dislike \| No opinion \| Like \| Strongly like \| \| --- \| --- \| --- \| --- \| --- \| \| **1** \| **2** \| **3** \| **4** \| **5** \|   How comfortable did you feel to engage in linkage to care intervention?   \| Very uncomfortable \| Uncomfortable \| No opinion \| Comfortable \| Very uncomfortable \| \| --- \| --- \| --- \| --- \| --- \| \| **1** \| **2** \| **3** \| **4** \| **5** \| | | | | | | |
|  | | |  | | | | | | |
| **Burden**  *The amount of effort required to participate in the intervention* | | | How much effort did it take to engage in the linkage to care intervention?   \| No effort at all \| A little effort \| No opinion \| A lot of effort \| Huge effort \| \| --- \| --- \| --- \| --- \| --- \| \| **1** \| **2** \| **3** \| **4** \| **5** \| | | | | | | |
|  | | |  | | | | | | |
| **Ethnicity**  *The extent to which the linkage to care intervention has good fit with an individual’s value system* | | | There are moral or ethical consequences to engage in the linkage to care intervention   \| Strongly disagree \| Disagree \| No opinion \| Agree \| Strongly agree \| \| --- \| --- \| --- \| --- \| --- \| \| **1** \| **2** \| **3** \| **4** \| **5** \|   How fair is the linkage to care intervention to people diagnosed with hypertension, diabetes of HIV?   \| Very unfair \| Unfair \| No opinion \| Fair \| Very fair \| \| --- \| --- \| --- \| --- \| --- \| \| **1** \| **2** \| **3** \| **4** \| **5** \| | | | | | | |
|  | | |  | | | | | | |
| **Perceived effectiveness**  *The extent to which the linkage to care intervention is have achieved its intended purpose* | | | The linkage to care intervention has improved my behaviour with my medical condition   \| Strongly disagree \| Disagree \| No opinion \| Agree \| Strongly agree \| \| --- \| --- \| --- \| --- \| --- \| \| **1** \| **2** \| **3** \| **4** \| **5** \| | | | | | | |
|  | | |  | | | | | | |
| **Intervention coherence**  *The extent to which the participant understands how the linkage to care intervention works* | | | The linkage to care intervention has improved my behaviour with my medical condition   \| Strongly disagree \| Disagree \| No opinion \| Agree \| Strongly agree \| \| --- \| --- \| --- \| --- \| --- \| \| **1** \| **2** \| **3** \| **4** \| **5** \| | | | | | | |
|  | | |  | | | | | | |
| **Self-efficacy**  *The participants confidence that they can perform behaviours required to participate in the linkage to care intervention* | | | How confident did you feel about engaging in the linkage to care intervention   \| Very not confident \| Fairly not confident \| No opinion \| Confident \| Very confident \| \| --- \| --- \| --- \| --- \| --- \| \| **1** \| **2** \| **3** \| **4** \| **5** \| | | | | | | |
|  | | |  | | | | | | |
| **Opportunity costs**  *The benefits, profits or values that were given up to engage in the linkage to care intervention* | | | The linkage to care intervention interfered with other priorities   \| Strongly disagree \| Disagree \| No opinion \| Agree \| Strongly agree \| \| --- \| --- \| --- \| --- \| --- \| \| **1** \| **2** \| **3** \| **4** \| **5** \| | | | | | | |
|  | | |  | | | | | | |
| **General acceptability** | | | How acceptable was the linkage to care intervention to you?   \| Very unacceptable \| Unacceptable \| No opinion \| Acceptable \| Very acceptable \| \| --- \| --- \| --- \| --- \| --- \| \| **1** \| **2** \| **3** \| **4** \| **5** \| | | | | | | |
|  | | |  | | | | | | |

## **SEDIRIŠWA SA KGOBOKETŠO YA DATHA SA KGOKAGANO YA BALWETŠI GO KAMOGELO YA TSENOGARE YA TLHOKOMELO BAKENG SA DIHLOPHA TŠA GO LATELA TŠA NAKO YE TELELE**

Hle araba dipotšišo tše tšeo di lebanego le dikarabelo tša gago lenaneong leo le šišintšwego la Kgokaganyo le Tlhokomelo. Dira sediko palo yeo e hlalosago dikarabelo tša gago gabotse.

| Sešupo sa Moswananoši sa Molwetši |  | | Boemo bja thuto | **Ga se ka tsena sekolo** | |  | Boemo bja lenyalo | **Single** |  |
| --- | --- | --- | --- | --- | --- | --- | --- | --- | --- |
| Mengwaga |  | |  | **Sekolo sa tlasana** | |  |  | **Nyetšwe** |  |
| Bong | **Monna** | **Mosadi** |  | **Sekolo sa bobedi** | |  |  | **Ba hlalane** |  |
| mohuta wa bolwetši |  | |  | **Sekolo sa boraro** | |  |  | **Mohlologadi** |  |
| Na o kalafong ya boemo bjo | **Ee** | **Aowa** | O na le nako ye kaakang o le kalafong (Duration in months or years) | |  | | | | |
|  |  |  | Ke ga kae o go kgoboketša dihlare | | **Kgwedi le kgwedi** | | **Kotara le kotara** | **Kgwedi ye tshela ka kgwedi** | |
| **Boemo bja kgopolo bjo bo nago le maikwelo**  *Kamoo motho ka noši a ikwago ka gona ka ga kgokagano le tsenogare ya tlhokomelo* | | | Na o ratile kgokagano ya tsenogare ya tlhokomelo   \| Go se rate ka matla \| Go se rate \| Ga go na kgopolo \| Key a Rata \| Rata ka Maatla \| \| --- \| --- \| --- \| --- \| --- \| \| **1** \| **2** \| **3** \| **4** \| **5** \|   Na o ratile kgokagano ya tsenogare ya tlhokomelo   \| Go se rate ka matla \| Go se rate \| Ga go na kgopolo \| Key a Rata \| Rata ka Maatla \| \| --- \| --- \| --- \| --- \| --- \| \| **1** \| **2** \| **3** \| **4** \| **5** \|   O ile wa ikwa o lokologile gakaakang go tsenela kgokagano le tsenogare ya tlhokomelo?   \| Ga se bohlokwa \| Ka tlase ga bohlokwa \| Ga go na kgopolo \| Bohlokwa \| Bohlokwa ka mo go feteletšego \| \| --- \| --- \| --- \| --- \| --- \| \| **1** \| **2** \| **3** \| **4** \| **5** \| | | | | | | |
|  | | |  | | | | | | |
| **Morwalo**  *Palo ya maitapišo ao a nyakegago go tšea karolo tsenogare* | | | Go tšere maiteko a makae go tsenela kgokagano le tsenogare ya tlhokomelo?   \| Ga go na maiteko le gatee \| Boiteko bjo bonyenyane \| Ga go na kgopolo \| Boiteko bjo bogolo \| Boiteko bjo bogolo kudu \| \| --- \| --- \| --- \| --- \| --- \| \| **1** \| **2** \| **3** \| **4** \| **5** \| | | | | | | |
|  | | |  | | | | | | |
| **Boitshwaro bja boitshwaro**  *Tekanyo yeo kgokagano le tsenogare ya tlhokomelo e nago le go swanela gabotse le tshepedišo ya boleng bja motho ka noši* | | | Go na le ditlamorago tša boitshwaro goba tša boitshwaro go tsenela kgokagano le tsenogare ya tlhokomelo   \| Ga ke dumelelane ka mo go feletšego \| Ga ke dumelelane le seo \| Ga go na kgopolo \| Key a Dumela \| Dumelelana ka matla \| \| --- \| --- \| --- \| --- \| --- \| \| **1** \| **2** \| **3** \| **4** \| **5** \|   Ke toka bjang kgokagano ya tsenogare ya tlhokomelo go batho bao ba lemogilwego gore ba na le kgatelelo ya madi, bolwetši bja swikiri bja HIV?   \| Go hloka toka kudu \| Go se loke \| Ga go na kgopolo \| Lokilego \| E lokile kudu \| \| --- \| --- \| --- \| --- \| --- \| \| **1** \| **2** \| **3** \| **4** \| **5** \| | | | | | | |
|  | | |  | | | | | | |
| **Go šoma gabotse mo go lemogwago**  *Tekanyo yeo kgokagano le tsenogare ya tlhokomelo e fihleletšego morero wa yona wo o bego o reretšwe* | | | Kgokagano le tsenogare ya tlhokomelo e kaonafaditše boitshwaro bja ka le boemo bja ka bja tša kalafo   \| Ga ke dumelelane ka mo go feletšego \| Ga ke dumelelane le seo \| Ga go na kgopolo \| Key a Dumela \| Dumelelana ka matla \| \| --- \| --- \| --- \| --- \| --- \| \| **1** \| **2** \| **3** \| **4** \| **5** \| | | | | | | |
|  | | |  | | | | | | |
| **Kgokagano ya tsenogare**  *Tekanyo yeo motšwasehlabelo a kwešišago ka moo kgokagano le tsenogare ya tlhokomelo e šomago ka gona* | | | Kgokagano le tsenogare ya tlhokomelo e kaonafaditše boitshwaro bja ka le boemo bja ka bja tša kalafo   \| Ga ke dumelelane ka mo go feletšego \| Ga ke dumelelane le seo \| Ga go na kgopolo \| Key a Dumela \| Dumelelana ka matla \| \| --- \| --- \| --- \| --- \| --- \| \| **1** \| **2** \| **3** \| **4** \| **5** \| | | | | | | |
|  | | |  | | | | | | |
| **Go ikgona**  *Batšeakarolo ba na le tshepo ya gore ba ka dira maitshwaro ao a nyakegago go tšea karolo kgokaganong ya tsenogare ya tlhokomelo* | | | O ile wa ikwa o itshepa gakaakang ka go tsenela kgokagano le tsenogare ya tlhokomelo   \| Go se ikholofele kudu \| Go se ikholofele \| Ga go na kgopolo \| Boitshepho \| E Itshepa kudu \| \| --- \| --- \| --- \| --- \| --- \| \| **1** \| **2** \| **3** \| **4** \| **5** \| | | | | | | |
|  | | |  | | | | | | |
| **Ditshenyagalelo tša sebaka**  *Mehola, dipoelo goba ditekanyetšo tšeo di ilego tša gafelwa go tsenela kgokagano le tsenogare ya tlhokomelo* | | | Kgokagano le tsenogare ya tlhokomelo e ile ya šitišana le dilo tše dingwe tše di tlago pele   \| Ga ke dumelelane ka mo go feletšego \| Ga ke dumelelane le seo \| Ga go na kgopolo \| Key a Dumela \| Dumelelana ka matla \| \| --- \| --- \| --- \| --- \| --- \| \| **1** \| **2** \| **3** \| **4** \| **5** \| | | | | | | |
|  | | |  | | | | | | |
| **Kamogelo ya kakaretšo** | | | Kgokagano ya tsenogare ya tlhokomelo e be e amogelega gakaakang go wena?   \| Ga e amogelege kudu \| Ga e amogelege \| Ga go na kgopolo \| E a amogelega \| E amogelega kudu \| \| --- \| --- \| --- \| --- \| --- \| \| **1** \| **2** \| **3** \| **4** \| **5** \| | | | | | | |
|  | | |  | | | | | | |

## **DATA COLLECTION TOOL TO COLLECT INFORMATION ON OVERALL COMFORT LEVELS WITH SHARING HEALTH DATA FOR RESEARCH AND POTENTIAL RISKS AND BENEFITS ASSOCIATED WITH HEALTH DATA SHARING**

| Patient Unique Identifier |  | | Educational level | | No schooling | | |  | Marital status | | Single | |  |
| --- | --- | --- | --- | --- | --- | --- | --- | --- | --- | --- | --- | --- | --- |
| Age in years |  | |  |  | Primary | | |  |  |  | Married | |  |
| Gender | **Male** | **Female** |  |  | Secondary | | |  |  |  | Divorced | |  |
| Medical condition |  | |  |  | Tertiary | | |  |  |  | Widowed | |  |
| Are you on treatment of this condition | **Yes** | **No** | For how long have you been on treatment (Duration in months or years) | | |  | | | | | | | |
|  |  |  | How often do you collect you medication | | | **Monthly** | | | **Quarterly** | | **Six-monthly** | | |
| 1. **How much would the removal of the following increase your comfort level with confidentially sharing your health data** | | | | | | | | | | | | | |
| Specific identifiers | | **Responses** | | | | | | | | | | | |
| Social security number | | Very uncomfortable | | Uncomfortable | | | No opinion | | | Comfortable | | Very Comfortable | |
| ID number | | Very uncomfortable | | Uncomfortable | | | No opinion | | | Comfortable | | Very Comfortable | |
| Home address | | Very uncomfortable | | Uncomfortable | | | No opinion | | | Comfortable | | Very Comfortable | |
| Date of birth | | Very uncomfortable | | Uncomfortable | | | No opinion | | | Comfortable | | Very Comfortable | |
| 1. **I am comfortable with my health data being confidentially shared with researchers, as long as personal information is not provided** | | | | | | | | | | | | | |
|  | | Completely Disagree | | Somewhat Disagree | | | Moderately Agree | | | Somewhat Agree | | Completely Agree | |
| 1. **I am comfortable with researchers not directly involved in my care accessing my electronic health data for research purposes** | | | | | | | | | | | | | |
|  | | Completely Disagree | | Somewhat Disagree | | | Moderately Agree | | | Somewhat Agree | | Completely Agree | |
| 1. **I am comfortable with someone I know (e.g., friend, neighbour, co-worker) who is a researcher accessing my electronic health data for research purposes** | | | | | | | | | | | | | |
|  | | Completely Disagree | | Somewhat Disagree | | | Moderately Agree | | | Somewhat Agree | | Completely Agree | |
| 1. **I am concerned about the potential risks associated with my health data being stolen by hackers** | | | | | | | | | | | | | |
|  | | Strongly disagree | | Disagree | | | No opinion | | | Agree | | Strongly agree | |
| 1. **I am concerned about the potential risks associated with my health data being re-identified and disclosed to employers** | | | | | | | | | | | | | |
|  | | Strongly disagree | | Disagree | | | No opinion | | | Agree | | Strongly agree | |
| 1. **I am concerned about the potential risks associated with my health data being re-identified and disclosed to people I know** | | | | | | | | | | | | | |
|  | | Strongly disagree | | Disagree | | | No opinion | | | Agree | | Strongly agree | |
| 1. **I am concerned about the potential risks associated with my health data being disclosed to researchers or doctors not involved in my care** | | | | | | | | | | | | | |
|  | | Strongly disagree | | Disagree | | | No opinion | | | Agree | | Strongly agree | |
| 1. **I am concerned about the potential risks associated with my health data being re-identified and disclosed to people I don’t know** | | | | | | | | | | | | | |
|  | | Strongly disagree | | Disagree | | | No opinion | | | Agree | | Strongly agree | |
| 1. **I believe the linkage to care intervention will help my nurses or doctors to make better decisions about my health** | | | | | | | | | | | | | |
|  | | Not important | | Less important | | | No opinion | | | Important | | Extremely important | |
| 1. **I believe the linkage to care intervention will help make new therapies available faster** | | | | | | | | | | | | | |
|  | | Not important | | Less important | | | No opinion | | | Important | | Extremely important | |
| 1. **I believe the linkage to care intervention will help researchers evaluate the quality of care delivered by nurses/doctors and clinics** | | | | | | | | | | | | | |
|  | | Not important | | Less important | | | No opinion | | | Important | | Extremely important | |
| 1. **I believe the linkage to care intervention will help other patients with improvement of their health condition** | | | | | | | | | | | | | |
|  | | Not important | | Less important | | | No opinion | | | Important | | Extremely important | |
| 1. **I believe the linkage to care intervention will help in reducing the cost of doing research** | | | | | | | | | | | | | |
|  | | Not important | | Less important | | | No opinion | | | Important | | Extremely important | |
| 1. **I believe the linkage to care intervention will help other patients with other health conditions** | | | | | | | | | | | | | |
|  | | Not important | | Less important | | | No opinion | | | Important | | Extremely important | |

## **Data collection tool to collect information on overall comfort levels with sharing health data for research and potential risks and benefits associated with health data sharing (Sepedi Version)**

| Sešupo sa Moswananoši sa Molwetši |  | | Boemo bja thuto | | **Ga se ka tsena sekolo** | | |  | Boemo bja lenyalo | | **Single** | |  |
| --- | --- | --- | --- | --- | --- | --- | --- | --- | --- | --- | --- | --- | --- |
| Mengwaga |  | |  |  | **Sekolo sa tlasana** | | |  |  |  | **Nyetšwe** | |  |
| Bong | **Monna** | **Mosadi** |  |  | **Sekolo sa bobedi** | | |  |  |  | **Ba hlalane** | | Bong |
| mohuta wa bolwetši |  | |  |  | **Sekolo sa boraro** | | |  |  |  | **Mohlologadi** | |  |
| Na o kalafong ya boemo bjo | **Ee** | **Aowa** | O na le nako ye kaakang o le kalafong (Duration in months or years) | | |  | | | | | | | |
|  |  |  | Ke ga kae o go kgoboketša dihlare | | | **Kgwedi le kgwedi** | | | **Kotara le kotara** | | **Kgwedi ye tshela ka kgwedi** | | |
| 1. Go tlošwa ga tše di latelago go be go tla oketša tekanyo ya gago ya boiketlo gakaakang ka go abelana ka sephiring ya data ya gago ya tša maphelo | | | | | | | | | | | | | |
| Specific identifiers | | **Responses** | | | | | | | | | | | |
| Nomoro ya faele ya molwetši | | Go sa phuthologa kudu | | Go sa phuthologa | | | Ga go na kgopolo | | | Phutholohile | | E phutholohile haholo | |
| Nomoro ya boitsibisho | | Go sa phuthologa kudu | | Go sa phuthologa | | | Ga go na kgopolo | | | Phutholohile | | E phutholohile haholo | |
| Aterese ya gae | | Go sa phuthologa kudu | | Go sa phuthologa | | | Ga go na kgopolo | | | Phutholohile | | E phutholohile haholo | |
| Letšatšikgwedi la matswalo | | Go sa phuthologa kudu | | Go sa phuthologa | | | Ga go na kgopolo | | | Phutholohile | | E phutholohile haholo | |
| 1. Ke phuthologile ka gore data ya ka ya maphelo e abelanwa ka sephiri le banyakišiši, ge fela tshedimošo ya motho e sa fiwe | | | | | | | | | | | | | |
|  | | Ga ke dumelelane ka mo go feletšego | | Ga ke dumelelane le seo | | | Ga go na kgopolo | | | Key a Dumela | | Dumelelana ka matla | |
| 1. Ke phuthologile ka banyakišiši bao ba sa amegego thwii tlhokomelong ya ka ba fihlelela datha ya ka ya maphelo ya elektroniki bakeng sa merero ya nyakišišo | | | | | | | | | | | | | |
|  | | Ga ke dumelelane ka mo go feletšego | | Ga ke dumelelane le seo | | | Ga go na kgopolo | | | Key a Dumela | | Dumelelana ka matla | |
| 1. Ke phuthologile le motho yo ke mo tsebago (mohlala, mogwera, moagišani, mošomi mmogo) yo e lego monyakišiši yo a fihlelelago datha ya ka ya maphelo ya elektroniki bakeng sa merero ya nyakišišo | | | | | | | | | | | | | |
|  | | Ga ke dumelelane ka mo go feletšego | | Ga ke dumelelane le seo | | | Ga go na kgopolo | | | Key a Dumela | | Dumelelana ka matla | |
| 1. Ke tshwenyegile ka dikotsi tšeo di ka bago gona tšeo di sepedišanago le go utswiwa ga data ya ka ya tša maphelo ke bahlakodi | | | | | | | | | | | | | |
|  | | Ga ke dumelelane ka mo go feletšego | | Ga ke dumelelane le seo | | | Ga go na kgopolo | | | Key a Dumela | | Dumelelana ka matla | |
| 1. Ke tshwenyegile ka dikotsi tšeo di ka bago gona tšeo di amanago le gore datha ya ka ya maphelo e lemogwe gape le go utollwa go bengmešomo | | | | | | | | | | | | | |
|  | | Ga ke dumelelane ka mo go feletšego | | Ga ke dumelelane le seo | | | Ga go na kgopolo | | | Key a Dumela | | Dumelelana ka matla | |
| 1. Ke tshwenyegile ka dikotsi tšeo di ka bago gona tšeo di sepedišanago le gore datha ya ka ya maphelo e lemogwe gape le go utollwa bathong bao ke ba tsebago | | | | | | | | | | | | | |
|  | | Ga ke dumelelane ka mo go feletšego | | Ga ke dumelelane le seo | | | Ga go na kgopolo | | | Key a Dumela | | Dumelelana ka matla | |
| 1. Ke tshwenyegile ka dikotsi tšeo di ka bago gona tšeo di sepedišanago le gore data ya ka ya tša maphelo e utollwe go banyakišiši goba dingaka tšeo di sa akaretšwago tlhokomelong ya ka | | | | | | | | | | | | | |
|  | | Ga ke dumelelane ka mo go feletšego | | Ga ke dumelelane le seo | | | Ga go na kgopolo | | | Key a Dumela | | Dumelelana ka matla | |
| 1. Ke tshwenyegile ka dikotsi tšeo di ka bago gona tšeo di amanago le gore datha ya ka ya maphelo e lemogwe gape le go utollwa bathong bao ke sa ba tsebego | | | | | | | | | | | | | |
|  | | Ga ke dumelelane ka mo go feletšego | | Ga ke dumelelane le seo | | | Ga go na kgopolo | | | Key a Dumela | | Dumelelana ka matla | |
| 1. Ke dumela gore kgokagano le tsenogare ya tlhokomelo e tla thuša baoki ba ka goba dingaka go tšea diphetho tše kaone ka ga maphelo a ka | | | | | | | | | | | | | |
|  | | Ga se bohlokwa | | Ka tlase ga bohlokwa | | | Ga go na kgopolo | | | Bohlokwa | | Bohlokwa ka mo go feteletšego | |
| 1. Ke dumela gore kgokagano le tsenogare ya tlhokomelo e tla thuša go dira gore dikalafo tše mpsha di hwetšagale ka lebelo | | | | | | | | | | | | | |
|  | | Ga se bohlokwa | | Ka tlase ga bohlokwa | | | Ga go na kgopolo | | | Bohlokwa | | Bohlokwa ka mo go feteletšego | |
| 1. Ke dumela gore kgokagano le tsenogare ya tlhokomelo e tla thuša banyakišiši go sekaseka boleng bja tlhokomelo yeo e fiwago ke baoki/dingaka le dikliniki | | | | | | | | | | | | | |
|  | | Ga se bohlokwa | | Ka tlase ga bohlokwa | | | Ga go na kgopolo | | | Bohlokwa | | Bohlokwa ka mo go feteletšego | |
| 1. Ke dumela gore kgokagano le tsenogare ya tlhokomelo e tla thuša balwetši ba bangwe ka kaonafatšo ya maemo a bona a maphelo | | | | | | | | | | | | | |
|  | | Ga se bohlokwa | | Ka tlase ga bohlokwa | | | Ga go na kgopolo | | | Bohlokwa | | Bohlokwa ka mo go feteletšego | |
| 1. Ke dumela gore kgokagano le tsenogare ya tlhokomelo e tla thuša go fokotša ditshenyegelo tša go dira nyakišišo | | | | | | | | | | | | | |
|  | | Ga se bohlokwa | | Ka tlase ga bohlokwa | | | Ga go na kgopolo | | | Bohlokwa | | Bohlokwa ka mo go feteletšego | |
| 1. Ke dumela gore kgokagano le tsenogare ya tlhokomelo e tla thuša balwetši ba bangwe bao ba nago le maemo a mangwe a maphelo | | | | | | | | | | | | | |
|  | | Ga se bohlokwa | | Ka tlase ga bohlokwa | | | Ga go na kgopolo | | | Bohlokwa | | Bohlokwa ka mo go feteletšego | |
