# Supplementary material for: Linkage to and retention in chronic care among patients diagnosed with hypertension, diabetes, or HIV in DIMAMO PHRC clinics, South Africa
Source: PLOS Glob Public Health. 2026 Feb 5;6(2):e0005362. doi: 10.1371/journal.pgph.0005362 (PMC12875484; doi:10.1371/journal.pgph.0005362)
Supplement: S1 Table — (DOCX) [file pgph.0005362.s002.docx]

**S1 Table: Number of patients diagnosed with either hypertension, diabetes, and or HIV per clinic linked to care**

| **Facility** | **Total in LCT** | **Excluded due to age below 18 years** | **Final population** | **Final Sample** |
| --- | --- | --- | --- | --- |
| Dikgale | 574 | 40 | 534 | 229 |
| Evelyn Lekganyane | 711 | 30 | 681 | 252 |
| J.Mamabolo | 430 | 18 | 412 | 203 |
| Makotopong | 514 | 26 | 488 | 220 |
| A.Mamabolo | 410 | 34 | 376 | 194 |
| Mamotshwa | 467 | 23 | 444 | 210 |
| Sebayeng | 539 | 11 | 528 | 228 |
| Mothiba | 271 | 3 | 268 | 160 |
| **GARND TOTAL** | **3916** | **185** | **3731** | **1696** |
