# Supplementary material for: Linkage to and retention in chronic care among patients diagnosed with hypertension, diabetes, or HIV in DIMAMO PHRC clinics, South Africa
Source: PLOS Glob Public Health. 2026 Feb 5;6(2):e0005362. doi: 10.1371/journal.pgph.0005362 (PMC12875484; doi:10.1371/journal.pgph.0005362)
Supplement: S2 Table — (DOCX) [file pgph.0005362.s003.docx]

**S2 Table: Distribution of Sample Size for patients diagnosed with hypertension, diabetes, and HIV per Facility**

| **Dikgale** | **Evelyn Lekganyane** | **J Mamabolo** | **Makotopong** |
| --- | --- | --- | --- |
| n = $\frac{534}{1+(0.05)^{2}}$  n =$\frac{534}{2.34}$  n=229 | n = $\frac{681}{1+(0.05)^{2}}$  n =$\frac{681}{2.702}$  n=252 | n = $\frac{412}{1+(0.05)^{2}}$  n =$\frac{412}{2.03}$  n=203 | n = $\frac{488}{1+(0.05)^{2}}$  n =$\frac{488}{2.22}$  n=220 |
| **Mamabolo A** | **Mamotswa** | **Mothiba** | **Sebayeng** |
| n = $\frac{376}{1+(0.05)^{2}}$  n =$\frac{376}{1.94}$  n=194 | n = $\frac{444}{1+(0.05)^{2}}$  n =$\frac{444}{2.11}$  n=210 | n = $\frac{268}{1+(0.05)^{2}}$  n =$\frac{268}{1.67}$  n=160 | n = $\frac{528}{1+(0.05)^{2}}$  n =$\frac{528}{2.32}$  n=228 |
