# Supplementary figures and images for: Linkage to and retention in chronic care among patients diagnosed with hypertension, diabetes, or HIV in DIMAMO PHRC clinics, South Africa
Source: PLOS Glob Public Health. 2026 Feb 5;6(2):e0005362. doi: 10.1371/journal.pgph.0005362 (PMC12875484; doi:10.1371/journal.pgph.0005362)

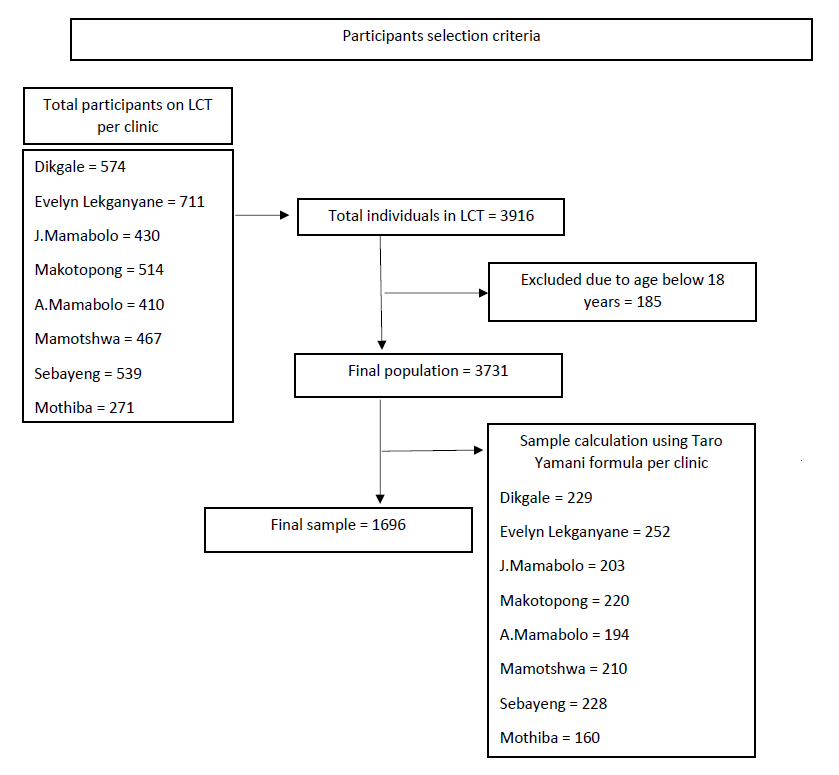


S1 Fig. Participants selection

Supplement: S1 Fig — (DOCX) [file pgph.0005362.s004.docx]
